# The Evaluation of the Antibacterial and Antifungal Efficacy of Novel Kids Mineral Trioxide Aggregate (e-MTA) With ProRoot MTA and Glass Ionomer Cement

**DOI:** 10.7759/cureus.27226

**Published:** 2022-07-25

**Authors:** Sourabh Joshi, Gowri Pendyala, Mukul Jain, Vinaya K Kulkarni, Swapnil Patil, Shantanu Choudhari

**Affiliations:** 1 Pediatric Dentistry, Rural Dental College, Pravara Institute of Medical Sciences, Loni, IND; 2 Periodontics, Rural Dental College, Pravara Institute of Medical Sciences, Loni, IND; 3 Periodontics, Kids-e Dental, Mumbai, IND; 4 Department of Pediatric Dentistry, Sau Mathurabai Bhausaheb Thorat (SMBT) Dental College and Hospital, Sangamner, IND; 5 Department of Pediatric and Preventive Dentistry, Mahatma Gandhi Vidyamandir’s Karmaveer Bhausaheb Hiray (KBH) Dental College, Nashik, IND; 6 Department of Periodontics, Government Dental College, Ahmedabad, IND

**Keywords:** glass ionomer cement, antifungal efficacy, antibacterial efficacy, proroot mta, e-mta

## Abstract

Background

Most pulpal and periapical problems could be treated nonsurgically. However, in cases of infections, certain operations must be performed that require using materials with good antibacterial and antifungal efficacy. ProRoot mineral trioxide aggregate (MTA) was marketed as gray- and white-colored preparations, composed of 75% Portland cement, 20% bismuth oxide, and 5% gypsum by weight. MTA, composed of powder and liquid as distilled water, formed a colloidal gel that further solidified and formed a hard cement within approximately four hours. The new endodontic material Kids e-MTA (Kids-e-dental, Mumbai, India) was introduced recently. It was also available as powder and liquid. It was a bioactive cement consisting of very fine hydrophilic particles of several mineral oxides.

Aim

This study compares the antimicrobial and antifungal efficacy of e-MTA (mineral trioxide aggregate) (Kids-e-dental, Mumbai, India), ProRoot MTA (Dentsply Sirona, Tulsa Dental, OK, USA), and glass ionomer cement (GIC) (GC Asia Dental Pte Ltd, Singapore).

Materials and methods

The agar diffusion method was used to test the materials. e-MTA, ProRoot MTA, and GIC were tested for their antibacterial efficacy against *Enterococcus faecalis* and antifungal efficacy against *Candida albicans*. The zone of inhibition was calculated and measured using a precision ruler. The collected data was put through Student’s unpaired t-test.

Results and conclusions

On conducting the tests and comparing the results, it was found that e-MTA had a slightly better antibacterial efficacy and almost similar antifungal efficacy compared to ProRoot MTA but significantly superior properties compared to GIC.

## Introduction

Microorganisms significantly impact the development and progression of pulpal and periapical diseases as well as failure of endodontic treatment [1]. The treatment’s success depends on the careful diagnosis and accurately performed cleaning and shaping, and a complete filling of the root canal. Most pulpal and periapical diseases were best managed nonsurgically. However, certain microorganisms were repeatedly recovered from previously root canal-filled teeth that had become infected. If nonsurgical endodontic therapy was unsuccessful, surgical endodontic therapy (apicoectomy and retrograde filling) is required to conserve the tooth. Most endodontic failures were attributable to inadequate root canal cleansing and ingressing bacteria and other antigens into the periradicular tissues. When infection or inflammation persists in the bony area around the end of the tooth after a root canal procedure, it is necessary to perform an apicoectomy. A root-end filling was placed to prevent reinfection of the root canal space [1,2]. Hence, in addition to sealing ability and biocompatibility, it was recommended that the root-end filling materials ideally should have some antimicrobial activity to prevent fungal and bacterial growth. One of the well-known root-end filling materials is glass ionomer cement (GIC), which had unique properties such as adhesion to the moist tooth structure, low shrinkage, and biological acceptance; however, it has low antimicrobial efficacy [3].

ProRoot mineral trioxide aggregate (MTA) is marketed as gray- and white-colored preparations, composed of 75% Portland cement, 20% bismuth oxide, and 5% gypsum by weight. MTA, composed of powder and liquid as distilled water, forms a colloidal gel that further solidifies and forms a hard cement within approximately four hours. Although MTA had excellent biocompatibility and antibacterial and antifungal efficacy, proving it to be the material of good standards, it had a delayed setting time and poor handling characteristics, as well as being an expensive material [4].

The new endodontic material Kids e-MTA (Kids-e-dental, Mumbai, India) is introduced recently. It is also available as powder and liquid. It is a bioactive cement that consists of very fine hydrophilic particles of several mineral oxides. The liquid consists of calcium chloride in an aqueous solution with an admixture of polycarboxylate, which sets in 12 minutes.

The manufacturer claims that the material e-MTA has good handling characteristics, quick setting time, high compressive strength, and good washout resistance. However, there were no studies or literature available proving these characteristics. Furthermore, the literature comparing the antibacterial and antifungal efficacy of e-MTA with its counterpart ProRoot MTA and glass ionomer cement is also scarce. Thus, the aim and objective of this study were to compare and investigate the antibacterial and antifungal effects of Kids e-MTA, ProRoot MTA, and GIC on *Enterococcus faecalis* and *Candida albicans*.

## Materials and methods

The study was performed at the Pravara Institute of Medical Sciences (PIMS), Loni, Ahmednagar, Maharashtra. It was an in vitro comparative study performed in the Department of Microbiology, and a trained microbiologist assisted in the study. Ethical clearance (PIMS/IEC-DR/2020/384) was obtained from the Institutional Ethical Committee of PIMS, Loni.

The test materials ProRoot MTA (Dentsply Sirona, Tulsa Dental, OK, USA), e-MTA (Kids-e-dental, Mumbai, India), and GIC (GC Asia Dental Pte Ltd, Singapore) were manipulated strictly in accordance with the manufacturer’s instructions and under strict aseptic precautions. *Enterococcus faecalis* ATCC 29212 and *Candida albicans* ATCC 10231 standard bacterial strains were used. The newer e-MTA was made available for the study purpose by Kids-e-dental, Mumbai. The endodontic cement’s antimicrobial activity was evaluated using the agar diffusion method. The experiment was performed using only test groups without positive and negative control groups. Each endodontic cement was evaluated at concentrations suggested by the manufacturer. The antimicrobial strains were diluted to obtain a suspension of approximately 0.5 McFarland, equal to 108 colony-forming units/mL, in sterile trypticase soy broth (TSB). *Enterococcus faecalis* and *C. albicans* suspensions were inoculated with sterile cotton swabs on Mueller-Hinton agar plates. Three wells, 4 mm in diameter and depth, were prepared on the plates with a copper puncher. These plates were filled with freshly manipulated test materials [5,6]. After pre-diffusion of the test materials for two hours at room temperature, all the plates were incubated at 37°C and evaluated at 24 hours. Microbial inhibition zones were measured with a 0.5-mm precision ruler, and the results were expressed as means and standard deviations (SD).

The data collected was compiled, tabulated, and subjected to comparative statistical analysis using SYSTAT version 12 (made by Crane’s software, Bangalore, India). A licensed copy was used to analyze the data. Statistical analysis was done by descriptive statistics such as mean, SD, percentage, and proportions. All assessment variables under study were compared by applying Student’s unpaired t-test at 5% (p = 0.05) and 1% (p = 0.01) levels of significance.

## Results

The results showed that e-MTA had the most potent antimicrobial activity with a zone of inhibition at 3.7 ± 0.97 against *E. faecalis*. In contrast, ProRoot MTA and GIC had a moderate antimicrobial activity with inhibition zones at 3.5 ± 1.07 and 1.4 ± 0.86. On intergroup comparison, it was evident that e-MTA was slightly better than ProRoot MTA but significantly superior to GIC. It could also be observed that ProRoot MTA and e-MTA had similar antimicrobial activity and inhibition zones at 2.60 ± 0.88 and 2.70 ± 0.99 against *C. albicans*, respectively. The confidence level selected was 95%. It was significantly greater than GIC at 1.30 ± 0.71 (Table 1).

**Table 1:** Mean inhibition zones of the three cement types (cm) MTA: mineral trioxide aggregate; GIC: glass ionomer cement; t: test value; p: probability value

Zone of inhibition	e-MTA (mean ± SD)	ProRoot MTA (mean ± SD)	Glass ionomer cement (mean ± SD)
Enterococcus faecalis	3.70 ± 0.97	3.50 ± 1.07	1.40 ± 0.86
Candida albicans	2.60 ± 0.88	2.70 ± 0.99	1.30 ± 0.71
Intra p-value	t = 1.205, p = 0.6874 (not significant)	t = 1.254, p = 0.7412 (not significant)	t = 0.8896, p = 0.5963 (not significant)

On comparing all three groups, it was clear that both MTA groups were superior to GIC (Table 2). On applying Student’s t-test, statistically significant results were seen between e-MTA and GIC for both *E. faecalis* and *C. albicans* (p = 0.003 and p = 0.002, respectively). A statistically significant value was also seen for ProRoot MTA and GIC (p = 0.04 and p = 0.006, respectively). e-MTA and ProRoot MTA did not show any statistically significant value for both *E. faecalis* and *C. albicans* (p = 0.90 and p = 0.92, respectively). The confidence level selected was 95%. It indicates that the antibacterial and antifungal activity of e-MTA and ProRoot MTA was similar. Both e-MTA and ProRoot MTA were superior to glass ionomer cement in terms of antimicrobial activity (Table 2).

**Table 2:** Comparative evaluation of the three cement types MTA: mineral trioxide aggregate; GIC: glass ionomer cement; t: test value; p: probability value

Zone of inhibition	Value of Student’s t-test and results		
	e-MTA versus ProRoot MTA	ProRoot MTA versus GIC	e-MTA versus GIC
Enterococcus faecalis	t = 1.07, p = 0.9022	t = 0.90, p = 0.04	t = 0.87, p = 0.0037
Candida albicans	t = 0.99, p = 0.92	t = 0.69, p = 0.006	t = 0.65, p = 0.002

## Discussion

Various materials have been suggested to seal the communication between the root canal system and the external environment. These materials were zinc phosphate, zinc oxide eugenol, intermediate restorative material (IRM), ethoxy benzoic acid (EBA), Cavit, and GIC. Unfortunately, none of these materials was ever ideal to be classed as repair materials. Therefore, MTA was developed in 1993, initially as experimental material. MTA was used for various operations, such as perforation repairs and apexification [7].

An essential property of root canal medicament was its antimicrobial and antifungal characteristics. When in close contact with tissue fluids, they should exhibit bactericidal and bacteriostatic effects, which would finally lead to tissue healing [5,6].

In our study, we considered the following materials: ProRoot MTA (Dentsply), e-MTA (Kids-e-dental), and GIC (GC Asia Dental Pte Ltd). MTA’s antibacterial and antifungal activity mainly functions as it contains calcium oxide that converts to calcium hydroxide when it comes in contact with tissue fluids. MTA’s antibacterial and antifungal activity was related to the increased pH at the site, leading to the deactivation and disruption of the microbial cellular membrane. On setting, MTA reaches a pH of 12.5. Therefore, its mechanism of action was bactericidal [6]. Earlier, GIC was used as it had properties such as adhesion to moist tooth surfaces, low shrinkage, fluoride release, and biocompatibility. It, however, had poor antimicrobial properties [8]. We have used the agar diffusion method, the most commonly used technique for evaluating antimicrobial activity [9]. This technique has been used by many authors in antimicrobial studies [10-12]. The bacteria chosen for our study were *E. faecalis* and *C. albicans* [13-15]. These bacteria were usually found in cases of endodontic failure and persistent infections.

There have not been many studies conducted on the antimicrobial efficacy of e-MTA. Our study was one of the few pioneering studies regarding the same. The agar diffusion method was used to prove the antimicrobial and antifungal efficacy of the test group. This method was one of the most familiar and standardized methods of proving antimicrobial and antifungal efficacies. In our study, we calculated the inhibition zones of each cement type. It was clear that both groups of MTA were superior compared to GIC. e-MTA was found to have a slightly more remarkable antimicrobial but an almost equal antifungal efficacy to ProRoot MTA.

The efficacy of ProRoot MTA being greater than GIC had already been proven in multiple previous studies conducted by various authors. Our results agreed with the study performed by Damlar et al. [8] and Bhavana et al. [16], in which they proved the antimicrobial properties of MTA against *E. faecalis*. In another study conducted by Al-Nazhan and Al-Judai [17], the antifungal properties of MTA were proved to be better, which was also proven by our study [17]. GIC was found to have poor antimicrobial and antifungal properties in both cases. Finally, in the study conducted by Estrela et al. [6] and Torabinejad et al. [9], MTA had no significant antimicrobial or antifungal properties, which were disproven by our study.

## Conclusions

MTA as a root-end filling material has proven effective due to its physical and chemical properties. The significant increase in its pH in the setting is attributed to its antimicrobial and antifungal properties. GIC was only used for its physical properties since its antimicrobial efficacy was poor. The new product e-MTA from Kids-e-dental had slightly better properties than ProRoot MTA and many superior properties compared to GIC. Further research should be conducted regarding the properties of e-MTA as it has the potential to be an efficient root-end filling material.
